# ODE-BPA: A Novel Basic Probability Assignment Generation Method Based on OTSU and Deng Entropy

**DOI:** 10.3390/e28060634

**Published:** 2026-06-03

**Authors:** Xinghua Zhou, Luyuan Chen, Enze Mao, Yutong He

**Affiliations:** College of Information Science and Technology & Artificial Intelligence, Nanjing Forestry University, Nanjing 210037, China; zhouxinghua777@gmail.com (X.Z.); 2310802114@njfu.edu.cn (E.M.); heyutong26@gmail.com (Y.H.)

**Keywords:** Dempster–Shafer theory, basic probability assignments, OTSU, Deng entropy, classification

## Abstract

Constructing basic probability assignments (BPAs) objectively and adaptively remains an important issue in Dempster–Shafer theory. To reduce the uncertainty caused by rigid nested focal element structures, this paper proposes ODE-BPA, a BPA generation method based on OTSU and Deng entropy. In ODE-BPA, hypothesis support values are normalized and sorted into an ordered sequence. OTSU is then used to construct two non-overlapping focal elements, while Deng entropy regulates the confidence degree for mass allocation. Experiments are conducted on twenty-five benchmark datasets, including mapping-structure comparison, ablation analysis, comparisons with existing BPA generation methods and classical machine learning classifiers, as well as the robustness evaluation under noisy and conflicting evidence. Rigorous statistical analysis demonstrates that ODE-BPA achieves competitive accuracy and ranking among existing BPA generation methods; exhibits performance comparable to that of SVM, naive Bayes, and decision tree under standard settings; and alleviates the influence of local noise and conflicting evidence in certain cases.

## 1. Introduction

Dempster–Shafer (D-S) theory is a well-established framework for reasoning under uncertainty [1,2,3]. Unlike classical Bayesian probability, it allows belief to be assigned not only to single hypotheses but also to subsets of the frame of discernment. This property makes D-S theory particularly suitable for representing epistemic uncertainty and ignorance. As a result, it has been widely applied in pattern classification [4,5,6], multi-sensor information fusion [7,8,9], fault diagnosis [10,11,12], emergency management [13,14] and risk assessment [15,16]. To address increasingly complex information environments, recent studies have further extended the D-S framework through various novel paradigms, such as Random Permutation Set theory [17,18,19,20], Quantum Evidence Theory [21,22,23], D numbers [24,25] and Complex Evidence Theory [26].

The effectiveness of D-S theory in practical applications critically depends on the construction of BPAs, because BPAs directly determine how uncertainty is quantified and propagated in evidential reasoning. In practice, BPA generation is often closely related to specific application scenarios and the characteristics of available data sources. Depending on the nature of the information, traditional generation methods are often subjective in nature and rely on domain experts to allocate belief masses. Although effective in some contexts, such approaches inevitably introduce human bias. To alleviate this limitation, researchers have explored scene- or model-based approaches that employ structural tools, such as confusion matrices [27,28,29] and fuzzy clustering [30,31,32], to construct BPAs. To further improve objectivity and adaptability, many studies have proposed data-sample-based methods. These data-driven approaches typically generate probability density functions or extract distribution characteristics directly from training samples. They usually employ either specific models, such as Gaussian distributions [33,34], or non-parametric estimation techniques [35,36,37]. By mining the intrinsic statistical properties of the data, these methods provide a rigorous and objective foundation for uncertainty modeling.

Notably, when transforming such continuous probability models into basic belief assignments, many data-sample-based methods adopt a nested mapping strategy [33,38,39]. A representative example is the Gaussian nested mapping method proposed by Xu et al. [33]. This method constructs class-conditional Gaussian distribution models from training data and evaluates a test sample by calculating its support degrees for different classes. These support degrees are then sorted in descending order and cumulatively assigned to focal elements, such as $C_{1},C_{1},C_{2},\ldots ,\Theta$, where Θ denotes the frame of discernment, i.e., the set of all candidate hypotheses. In this way, lower-ranked hypotheses are not assigned to independent focal elements. Instead, they are absorbed into supersets that already contain higher-ranked hypotheses, which results in a nested BPA structure. This framework has been widely adopted and has inspired several extensions. For example, Xu et al. [35] replaced Gaussian models with non-parametric probability density functions while retaining the nested structure. Fu et al. [40] further introduced a method based on normal membership functions to better characterize data boundaries. Despite these developments, most existing methods still rely on a strictly nested focal element structure.

However, the Gaussian nested mapping strategy still suffers from some limitations. Once the BPA is constrained to a nested focal element configuration, strong support for the leading hypothesis cannot be maintained independently throughout the assignment process, but is instead progressively propagated into larger supersets containing lower-ranked alternatives. This effect becomes particularly problematic in conflicting environments. Consider a classification problem with three hypotheses, *A*, *B*, and *C*, where *A* receives much stronger support than *B* and *C*. A nested mapping method typically generates focal elements such as {A}, {A,B}, and {A,B,C}. In this case, the dominant hypothesis *A* is repeatedly grouped with weaker or even conflicting hypotheses in subsequent focal elements. Consequently, the discriminative role of the strongest evidence may be weakened, and excessive belief mass may be allocated to the universal set {A,B,C}. As a result, the induced BPA may become less informative for subsequent evidential reasoning and classification.

To address the above limitations, this paper proposes a novel BPA generation method based on OTSU and Deng Entropy, termed ODE-BPA. The proposed method combines OTSU [41] with Deng entropy [42] to construct focal elements and allocate belief masses in a more adaptive manner. Unlike nested mapping methods, OTSU does not force the hypotheses into a chain of inclusive subsets. Instead, it identifies an objective partition by maximizing the between-group variance of the ordered support degrees. In this way, hypotheses with relatively strong support can be grouped into one subset, whereas those with weaker support are assigned to another subset. The resulting focal elements are therefore distinct and non-overlapping, rather than successively embedded into one another. Based on this partition, the support degrees within each group are aggregated to form preliminary mass values, after which Deng entropy is introduced to quantify the uncertainty of the resulting focal element structure. Since Deng entropy takes into account not only the distribution of masses but also the cardinalities of focal elements, it can characterize both the discord among hypotheses and the non-specificity of subsets [43]. In this manner, ODE-BPA uses OTSU to construct a data-driven grouping structure and employs Deng entropy to regulate how confidently the grouped evidence should be represented in the final BPA.

The main contributions of this work are threefold. First, OTSU is introduced for focal element construction, thereby alleviating the structural rigidity imposed by conventional nested BPA generation schemes. Second, an uncertainty-aware mass allocation strategy is developed, in which the confidence coefficient is determined by the relationship between the Deng entropy and the maximum Deng entropy. Third, extensive experiments are conducted on twenty-five benchmark datasets, including internal ablation analysis, comparisons with existing BPA generation methods, comparisons with classical machine learning classifiers, and robustness evaluations under noisy and conflicting evidence. The results show that the proposed ODE-BPA method performs competitively among existing BPA generation methods. In comparison with classical machine learning classifiers, it provides a statistically comparable evidential classification alternative to SVM, naive Bayes, and decision tree under standard settings, rather than showing clear superiority over all classifiers.

The remainder of this paper is organized as follows. Section 2 reviews the basic principles of D-S theory, Deng entropy, and OTSU. Section 3 presents the mathematical formulation of the proposed ODE-BPA method. Section 4 provides a numerical illustration. Section 5 reports the experimental results on benchmark datasets. Finally, Section 6 concludes the paper.

## 2. Preliminaries

This section reviews the theoretical background of the proposed method. It introduces the basic concepts of D-S theory, Deng entropy, and OTSU.

### 2.1. D-S Theory

Uncertainty is a fundamental issue in real-world reasoning and decision-making [44,45,46]. Dempster–Shafer (D-S) theory, as introduced by Dempster and then developed by Shafer, has emerged as a rigorous mathematical framework by generalizing the classic probability theory [47,48]. To facilitate the subsequent presentation of the proposed method, the basic concepts of D-S theory are briefly reviewed below.

Let $\Theta =\{\theta_{1},\theta_{2},\ldots ,\theta_{n}\}$ denote a finite set of mutually exclusive and collectively exhaustive hypotheses, called the frame of discernment. Its power set $2^{\Theta }$ consists of all subsets of Θ. A basic probability assignment (BPA), also called a mass function *m*, is a mapping $m:2^{\Theta }\to \left[0,1\right]$ satisfying the following conditions [49,50]:

(1) $$m\left(\emptyset \right)=0,\;\sum_{A\subseteq \Theta }m\left(A\right)=1.$$

Here, m(A) denotes the belief assigned exactly to proposition *A*. A subset *A* is called a focal element if m(A)>0. When two independent bodies of evidence are defined on the same frame of discernment Θ, they can be combined by Dempster’s rule of combination. For two mass functions $m_{1}$ and $m_{2}$, their orthogonal sum $m=m_{1}⊕m_{2}$ is given by [49,50]:

(2) $$m\left(A\right)=\frac{1}{1-K}\sum_{B\cap C=A}m_{1}\left(B\right)m_{2}\left(C\right),\;A\neq \emptyset ,$$

where the normalization factor *K* is defined as [49,50]:

(3) $$K=\sum_{B\cap C=\emptyset }m_{1}\left(B\right)m_{2}\left(C\right).$$

The quantity *K* is called the conflict coefficient, measuring the degree of inconsistency between pieces of evidence.

After evidence combination, a decision-making rule is usually required to select the final hypothesis. In this paper, the pignistic probability transformation (PPT), proposed by Smets and Kennes within the transferable belief model, is adopted to convert a BPA into a probability distribution for decision making [51]. The basic idea of PPT is to redistribute the mass assigned to each non-singleton focal element equally among the singleton hypotheses contained in that focal element. For any singleton hypothesis $\theta_{i}\in \Theta$, its pignistic probability is defined as [51]

(4) $$BetP\left(\theta_{i}\right)=\sum_{\begin{array}{c} A\subseteq \Theta \\ \theta_{i}\in A \end{array}}\frac{m(A)}{\left|A\right|},$$

where |A| denotes the cardinality of focal element *A*. Since m(∅)=0 is maintained in the standard D-S framework considered in this paper, no additional normalization term is needed. The final decision is then made by selecting the hypothesis with the largest pignistic probability, namely

(5) $$\theta^{*}=\arg\max_{\theta_{i}\in \Theta }BetP\left(\theta_{i}\right).$$

This transformation provides a convenient bridge between belief assignment and probabilistic decision making, and is therefore used in the experimental classification stage of this study.

### 2.2. Deng Entropy

As a fundamental physical concept, entropy has been widely adopted for uncertainty quantification [52,53,54,55,56]. However, within evidence theory, uncertainty exhibits a more intricate structure, involving both nonspecificity and discord. To characterize this multifaceted evidential uncertainty, Deng entropy has been proposed for basic probability assignments [42]. This measure represents an important attempt to quantify uncertainty in evidence theory and has subsequently attracted significant research attention. Existing studies have investigated its theoretical properties and practical applications, including the linear characteristic [57], the physical interpretation from the perspective of information coding [58], and a variety of application models developed based on Deng entropy [59].

For a mass function *m* defined on a frame of discernment Θ, Deng entropy $H_{d}$ is defined as [42]:

(6) $$H_{d}=-\sum_{A\subseteq \Theta }m\left(A\right)\log_{2}\frac{m(A)}{2^{\left|A\right|}-1},$$

where |A| denotes the cardinality of subset *A*. Compared with Shannon entropy, which is defined over singleton hypotheses, Deng entropy introduces the term $\left(2^{\left|A\right|}-1\right)$ to account for the additional uncertainty associated with multi-element focal elements. When all focal elements are singletons, Deng entropy reduces to Shannon entropy.

For a fixed focal element set F, Kang and Deng [60] derived the maximum Deng entropy, which occurs when the mass assignment satisfies $m\left(A\right)\propto \left(2^{\left|A\right|}-1\right)$ for each A∈F. Under this condition, the maximum Deng entropy $H_{\max}$ is given by [60]:

(7) $$H_{\max}=\log_{2}\left(\sum_{A\in F}\left(2^{\left|A\right|}-1\right)\right).$$

The value $H_{\max}$ provides a structure-dependent upper bound for Deng entropy under a given focal element configuration.

### 2.3. OTSU

OTSU [41] is a classical nonparametric threshold selection method originally developed for image segmentation. Its basic idea is to determine a threshold that maximizes the between-class variance. In this way, the data can be divided into two groups with the greatest statistical separation, without requiring manual parameter tuning.

Assume that a dataset is divided by a candidate threshold *t* into two classes, denoted by $C_{1}$ and $C_{2}$. Let $\omega_{1}\left(t\right)$ and $\omega_{2}\left(t\right)$ denote the proportions of the two classes, with $\omega_{1}\left(t\right)+\omega_{2}\left(t\right)=1$. Let $\mu_{1}\left(t\right)$ and $\mu_{2}\left(t\right)$ denote their corresponding mean values. The between-class variance is defined as [41]

(8) $$\sigma_{B}^{2}\left(t\right)=\omega_{1}\left(t\right)\omega_{2}\left(t\right){\left(\mu_{1}\left(t\right)-\mu_{2}\left(t\right)\right)}^{2}.$$

The optimal threshold $t^{*}$ is obtained by maximizing the between-class variance:

(9) $$t^{*}=\arg\max_{t}\sigma_{B}^{2}\left(t\right).$$

Because OTSU is fully data-driven and does not rely on manually specified thresholds, its variance-maximization principle can be extended beyond image processing. In this work, OTSU is used to partition an ordered evidential sequence. This produces an automatic and objective grouping of hypotheses, which serves as the structural basis for focal element construction in the proposed BPA generation method.

## 3. The Proposed ODE-BPA Method

**Figure 1:**
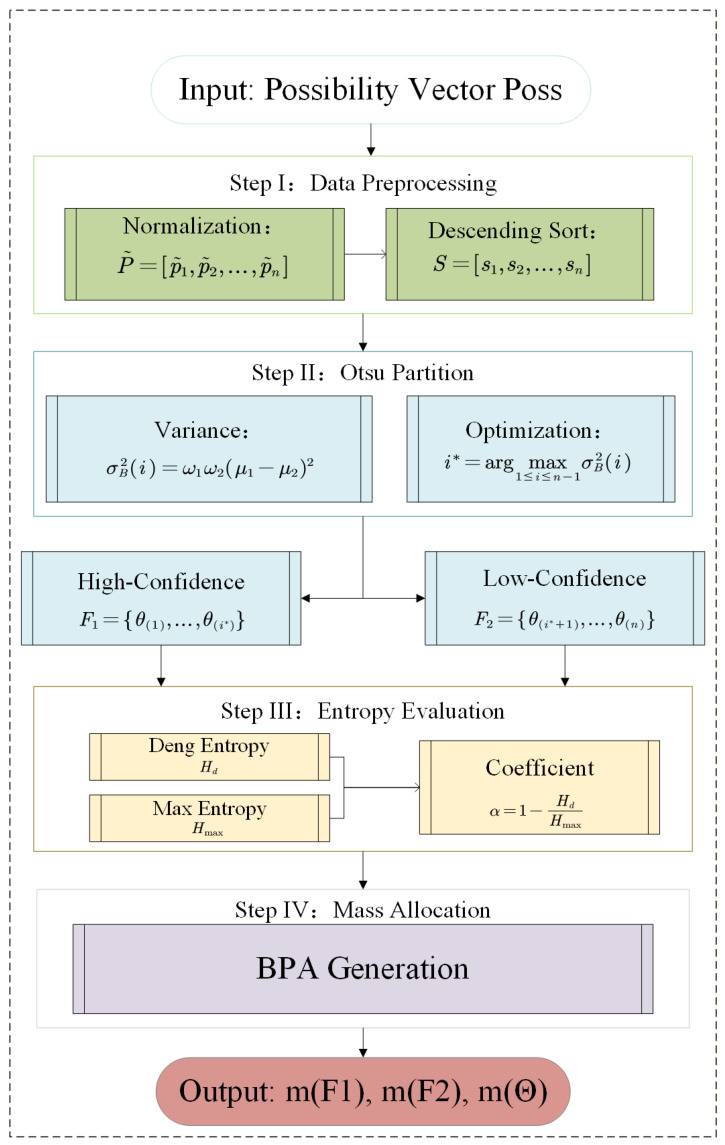
Framework of the proposed ODE-BPA method. The superscript ∗ indicates the optimal split index determined by the OTSU algorithm.

To address the limitations inherent in conventional BPA construction techniques—particularly the structural rigidity imposed by nested mapping schemes—this paper introduces a novel evidential modeling approach termed ODE-BPA, which seamlessly integrates the OTSU algorithm with Deng entropy. This method is designed to adaptively construct focal elements based on the relative support patterns of hypotheses, and to subsequently regulate belief mass allocation guided by the uncertainty of the induced evidential structure. The proposed framework encompasses four primary stages: evidential support modeling, focal element construction, uncertainty quantification, and confidence-based mass assignment. The overall architecture is depicted in Figure 1.

### 3.1. Evidential Support Modeling

Consider a classification problem defined on a frame of discernment

$$\Theta =\{\theta_{1},\theta_{2},\ldots ,\theta_{n}\}$$

within the D-S theory framework. For a given feature *f* of an input sample *x*, the support degree associated with hypothesis $\theta_{j}$ is estimated by the Gaussian probability density function [33]

(10) $$p_{j}=\frac{1}{\sqrt{2\pi }\sigma_{j,f}}\exp\left(-\frac{{\left(x_{f}-\mu_{j,f}\right)}^{2}}{2\sigma_{j,f}^{2}}\right)$$

where $\mu_{j,f}$ and $\sigma_{j,f}$ denote the mean and standard deviation of the *f*-th feature under class $\theta_{j}$, respectively, estimated from the training data. The Gaussian density is employed herein as a pragmatic statistical model for continuous features, yielding an initial measure of relative support for each hypothesis.

It is crucial to note that the ODE-BPA framework is not strictly bound to this specific distributional assumption. The Gaussian model merely serves to initialize the support representation. The core of the proposed method operates on the ordered rank-support patterns derived from these initial values.

The resulting support vector is written as

$$P=[p_{1},p_{2},\ldots ,p_{n}].$$

To obtain a normalized evidential representation, the support values are transformed as

(11) $${\tilde{p}}_{j}=\frac{p_{j}}{\sum_{t=1}^{n}p_{t}},\;j=1,2,\ldots ,n.$$

This normalization yields the support distribution

$$\tilde{P}=\left[{\tilde{p}}_{1},{\tilde{p}}_{2},\ldots ,{\tilde{p}}_{n}\right].$$

Sorting the normalized support values in descending order gives the ordered evidential sequence

$$S=\left[s_{1},s_{2},\ldots ,s_{n}\right],\;s_{1}\geq s_{2}\geq \ldots \geq s_{n}.$$

The corresponding hypotheses are rearranged accordingly as

$$\Theta_{S}=\left\{\theta_{\left(1\right)},\theta_{\left(2\right)},\ldots ,\theta_{\left(n\right)}\right\}.$$

### 3.2. Focal Element Construction via OTSU

To construct focal elements from the ordered evidential sequence *S*, the one-dimensional OTSU algorithm is utilized. For a candidate split index *i* (1≤i≤n−1), the sequence is partitioned into two groups

$$\text{High-Confidence}\;\mathrm{Group}=\left\{s_{1},\ldots ,s_{i}\right\},\;\text{Low-Confidence}\;\mathrm{Group}=\left\{s_{i+1},\ldots ,s_{n}\right\}.$$

The corresponding proportions are

$$\omega_{1}=\frac{i}{n},\;\omega_{2}=\frac{n-i}{n}.$$

The between-group variance associated with split index *i* is defined as

(12) $$\sigma_{B}^{2}\left(i\right)=\omega_{1}\omega_{2}{\left(\mu_{1}-\mu_{2}\right)}^{2}$$

where

$$\mu_{1}=\frac{1}{i}\sum_{t=1}^{i}s_{t},\;\mu_{2}=\frac{1}{n-i}\sum_{t=i+1}^{n}s_{t}.$$

The optimal split index is then uniquely determined by maximizing the between-group variance:

(13) $$i^{*}=\arg\max_{1\leq i\leq n-1}\sigma_{B}^{2}\left(i\right).$$

Accordingly, the reordered hypotheses are partitioned into two distinct focal elements

$$F_{1}=\left\{\theta_{\left(1\right)},\ldots ,\theta_{\left(i^{*}\right)}\right\},\;F_{2}=\left\{\theta_{\left(i^{*}+1\right)},\ldots ,\theta_{\left(n\right)}\right\}.$$

In stark contrast to conventional nested mapping paradigms, this formulation does not coerce hypotheses into a rigid chain of inclusive subsets. Instead, it dynamically derives two mutually exclusive focal elements directly from the rank-ordered evidential support, thereby facilitating a highly adaptive focal element architecture.

### 3.3. Uncertainty Quantification via Deng Entropy

Once the focal elements are determined, the preliminary masses associated with them are obtained by aggregating the support values within each corresponding group:

$$m_{1}=\sum_{t=1}^{i^{*}}s_{t},\;m_{2}=\sum_{t=i^{*}+1}^{n}s_{t}.$$

Their respective cardinalities are

$$C_{1}=|F_{1}|=i^{*},\;C_{2}=\left|F_{2}\right|=n-i^{*}.$$

The uncertainty inherent in the induced focal element structure is then quantified utilizing Deng entropy:

(14) $$H_{d}=-m_{1}\log_{2}\frac{m_{1}}{2^{C_{1}}-1}-m_{2}\log_{2}\frac{m_{2}}{2^{C_{2}}-1}.$$

To normalize this uncertainty measure, the corresponding maximum Deng entropy is introduced:

(15) $$H_{\max}=\log_{2}\left(\left(2^{C_{1}}-1\right)+\left(2^{C_{2}}-1\right)\right).$$

Here, $H_{d}$ quantifies the empirical uncertainty inherent in the derived two-focal-element structure, while $H_{\max}$ establishes its theoretical upper bound given the same cardinality configuration. The ratio between these two metrics serves as a critical regulator for determining the confidence level in the subsequent mass assignment phase.

### 3.4. BPA Construction via Confidence-Based Mass Assignment

Drawing upon the relationship between the empirical uncertainty $H_{d}$ and its theoretical maximum $H_{\max}$, a confidence coefficient α is formulated as:

(16) $$\alpha =1-\frac{H_{d}}{H_{\max}}.$$

This coefficient acts as a direct proxy for the reliability of the constructed focal element structure. A lower uncertainty yields a higher α, indicating that a greater proportion of the belief mass should be committed to the derived focal elements. Conversely, elevated uncertainty results in a diminished α, thereby reserving more mass for the global frame of discernment Θ to adequately capture residual ignorance.

Consequently, the final BPA is generated as follows:

(17) $$m\left(A\right)=\left\{\begin{array}{cc} \alpha m_{1}, & A=F_{1},\\ \alpha m_{2}, & A=F_{2},\\ 1-\alpha , & A=\Theta , \end{array}\right.$$

where m(Θ) denotes the uncommitted belief allocated to the frame of discernment. Through this mechanism, the ODE-BPA method formalizes a comprehensive basic probability assignment, synergizing data-driven focal element derivation with entropy-regulated confidence calibration. The complete algorithmic procedure is summarized in Algorithm 1.

**Algorithm 1** The Proposed ODE-BPA Method**Require:** Feature value $x_{f}$ of an input sample;  Frame of discernment $\Theta =\{\theta_{1},\theta_{2},\ldots ,\theta_{n}\}$;  Mean $\mu_{j,f}$ and standard deviation $\sigma_{j,f}$ for j=1,…,n.**Ensure:** Basic Probability Assignment m(·).
 1:
/* Stage 1: Evidential Support Modeling */
 2:Compute initial support vector $P=[p_{1},\ldots ,p_{n}]$ using Equation (10) 3:Normalize *P* to obtain $\tilde{P}=\left[{\tilde{p}}_{1},\ldots ,{\tilde{p}}_{n}\right]$ (Equation (11)) 4:Sort $\tilde{P}$ in descending order to get sequence *S* and reordered hypotheses $\Theta_{S}$ 5:
/* Stage 2: Focal Element Construction via OTSU */
 6:**for** i=1 **to** n−1 **do** 7:  Compute between-group variance $\sigma_{B}^{2}\left(i\right)$ (Equation (12)) 8:
**end for**
 9:Select optimal split $i^{*}=\arg\max_{i}\sigma_{B}^{2}\left(i\right)$ (Equation (13)) 10:Partition $\Theta_{S}$ into $F_{1}=\left\{\theta_{\left(1\right)},\ldots ,\theta_{\left(i^{*}\right)}\right\}$, $F_{2}=\left\{\theta_{\left(i^{*}+1\right)},\ldots ,\theta_{\left(n\right)}\right\}$ 11:
/* Stage 3: Uncertainty Quantification via Deng Entropy */
 12:Aggregate preliminary masses: $m_{1}=\sum_{t=1}^{i^{*}}s_{t}$, $m_{2}=\sum_{t=i^{*}+1}^{n}s_{t}$ 13:Compute empirical Deng entropy $H_{d}$ (Equation (14)) and maximum Deng entropy $H_{\max}$ (Equation (15)) 14:Calculate confidence coefficient $\alpha =1-H_{d}/H_{\max}$ 15:
/* Stage 4: Mass Assignment */
 16:Assign belief masses:$m\left(F_{1}\right)=\alpha \cdot m_{1},\;m\left(F_{2}\right)=\alpha \cdot m_{2},\;m\left(\Theta \right)=1-\alpha$ 17:**return** m(·)


### 3.5. Computational Complexity Analysis

To evaluate the computational efficiency of the proposed ODE-BPA method, let *n* denote the number of hypotheses in the frame of discernment Θ. The algorithmic runtime is determined by its four main stages: (1) Evidential Support Modelingrequires O(n) time to compute and normalize the probabilities. (2) Sorting Operation takes O(nlogn) time to reorder the sequence. (3) OTSU Partition can be strictly executed in O(n) time rather than the naive $O(n^{2})$; by maintaining cumulative sums of weights and means, the variance update at each candidate split is performed in O(1) time. (4) Uncertainty Quantification and Mass Assignment involve only constant-time scalar arithmetic, thus running in O(1) time.

Consequently, the overall time complexity of ODE-BPA is exclusively bounded by the sorting stage at O(nlogn). Given that the cardinality *n* is generally small in standard classification tasks, the proposed method guarantees high computational efficiency and practical scalability.

## 4. Numerical Example

To illustrate the proposed ODE-BPA method more clearly and compare it with the existing method, numerical examples are provided under both a general case and a special highly uncertain case.

Assume the frame of discernment is $\Theta =\{\theta_{1},\theta_{2},\theta_{3},\theta_{4}\}$ and the initial support vector associated with a target sample is given by

P=[0.195,0.104,0.936,0.065].

It can be seen that the elements of *P* are neither normalized nor ordered. Therefore, the first step of ODE-BPA is to normalize the raw support values. Since $\sum_{j=1}^{4}p_{j}=1.3$, the normalized support distribution is obtained as

$$\tilde{P}=\left[0.15,0.08,0.72,0.05\right].$$

Next, the normalized support values are sorted in descending order, yields the ordered evidential sequence

S=[0.72,0.15,0.08,0.05].

Accordingly, the associated hypotheses are reordered as

$$\Theta_{S}=\left\{\theta_{3},\theta_{1},\theta_{2},\theta_{4}\right\},$$

meaning $\theta_{\left(1\right)}=\theta_{3}$, $\theta_{\left(2\right)}=\theta_{1}$, $\theta_{\left(3\right)}=\theta_{2}$, and $\theta_{\left(4\right)}=\theta_{4}$.

According to the OTSU partitioning strategy, the candidate split indices are i∈{1,2,3}. The corresponding between-group variances are computed as

$$\sigma_{B}^{2}\left(1\right)\approx 0.0737,\;\sigma_{B}^{2}\left(2\right)\approx 0.0342,\;\sigma_{B}^{2}\left(3\right)\approx 0.0134.$$

**Figure 2:**
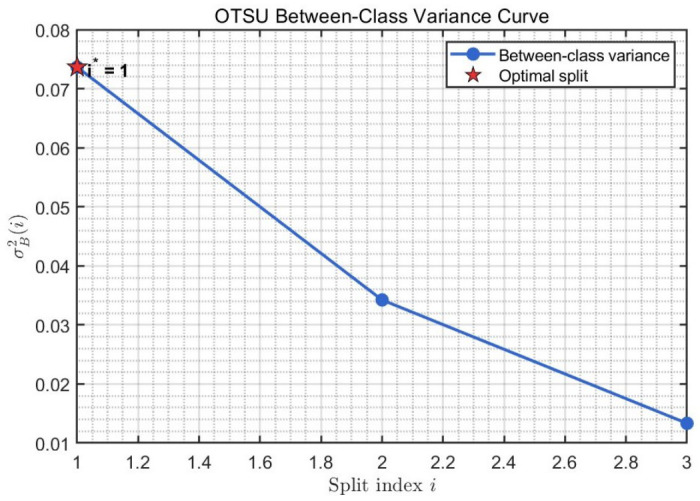
Between-group variance curve of the OTSU criterion with respect to the split index. The ∗ indicates the maximum between-group variance corresponding to the optimal split index.

As shown in Figure 2, the maximum variance is attained at $i^{*}=1$. The reordered hypotheses are thus partitioned into two focal elements:

$$F_{1}=\left\{\theta_{\left(1\right)}\right\}=\left\{\theta_{3}\right\},\;F_{2}=\left\{\theta_{\left(2\right)},\theta_{\left(3\right)},\theta_{\left(4\right)}\right\}=\left\{\theta_{1},\theta_{2},\theta_{4}\right\}.$$

The corresponding aggregated masses and cardinalities are $m_{1}=0.72,\;C_{1}=1$ and $m_{2}=0.28,\;C_{2}=3$.

Based on these two focal elements, the Deng entropy of the induced evidential structure is calculated as $H_{d}=1.641$. Under the same cardinality configuration, the maximum Deng entropy is $H_{\max}=3.000$. The confidence coefficient is given by

$$\alpha =1-\frac{H_{d}}{H_{\max}}=0.453.$$

Finally, the BPA generated by ODE-BPA is

$$\begin{array}{cc} m(\left\{\theta_{3}\right\}) & =0.3262,\\ m(\left\{\theta_{1},\theta_{2},\theta_{4}\right\}) & =0.1268,\\ m(\Theta ) & =0.5470. \end{array}$$

For comparison, we apply Xu’s method [33] to the same initial vector *P*, treating it as the normal distribution intersection values. Sorted in descending order, the values are $w_{1}=0.936$, $w_{2}=0.195$, $w_{3}=0.104$, and $w_{4}=0.065$, associated with $\theta_{3}$, $\theta_{1}$, $\theta_{2}$, and $\theta_{4}$, respectively. According to Xu’s rule, the nested BPA before normalization is assigned as

$$m\left(\left\{\theta_{3}\right\}\right)=0.936,\;m\left(\left\{\theta_{3},\theta_{1}\right\}\right)=0.195,$$

$$m\left(\left\{\theta_{3},\theta_{1},\theta_{2}\right\}\right)=0.104,\;m\left(\Theta \right)=0.065.$$

After dividing by the sum 1.3, Xu’s final BPA is

$$\begin{array}{cc} m_{Xu}\left(\left\{\theta_{3}\right\}\right) & =0.7200,\\ m_{Xu}\left(\left\{\theta_{3},\theta_{1}\right\}\right) & =0.1500,\\ m_{Xu}\left(\left\{\theta_{3},\theta_{1},\theta_{2}\right\}\right) & =0.0800,\\ m_{Xu}\left(\Theta \right) & =0.0500. \end{array}$$

To directly compare the uncertainty characteristics of the two BPA generation strategies, Table 1 reports the generated BPA structures together with their entropy. As shown in Table 1, the traditional nested mapping method generates several nested focal elements containing the dominant hypothesis $\theta_{3}$. In contrast, ODE-BPA keeps $\theta_{3}$ as an independent singleton focal element and places the remaining hypotheses into a separate non-overlapping focal element. This leads to a lower Deng entropy of the focal structure, indicating a more compact representation of the dominant evidence. It is also noted that ODE-BPA assigns a larger mass to Θ. This does not mean that the dominant evidence is weakened. Instead, it reflects the Deng-entropy-based confidence regulation mechanism, which transfers uncertain residual belief to global ignorance rather than forcing it into nested focal elements. Therefore, the proposed method provides a more cautious and structurally interpretable BPA while preserving the dominant hypothesis.

## 5. Experimental Validations

To comprehensively evaluate the classification performance, stability, and robustness of the proposed ODE-BPA method, and to ensure high statistical reliability as recommended for multiple classifier comparisons [61], extensive experiments are conducted on twenty-five benchmark datasets. These datasets include Cryotherapy, Immunotherapy, Acute Inflammations, Divorce, Planning, Fire, Haberman, Vertebral Column (2C), Mesothelioma, Voting, Wholesale Customers, Early Diabetes, Monk1, WDBC, Australian, Blood Transfusion, Fourclass, Raisin, Banknote, Lenses, Iris, Wine, Seeds, Soybean, and Knowledge. All of these datasets are obtained from the UCI Machine Learning Repository. The Fire dataset is derived from the Algerian forest fires dataset, where the region attribute is removed during preprocessing. Therefore, the remaining attributes are used for classification. For the other datasets, no prior data preprocessing is performed. The basic information of the datasets used in the experiments is summarized in Table 2. These datasets cover different numbers of samples, features, and classes, which provides a diverse experimental environment for evaluating the effectiveness of different BPA generation methods.

To reduce the influence of random data partitioning and obtain statistically reliable results, all experiments are independently repeated 100 times. In each independent run, a stratified five-fold cross-validation strategy is adopted. The classification accuracy is first averaged over the folds in each run, and the final mean accuracy and standard deviation are then calculated over 100 independent runs. Since one class in the Lenses dataset contains only four samples, four-fold cross-validation is used for this dataset instead of five-fold cross-validation to ensure that each fold contains valid class information.

The experimental validation is organized into six parts: comparison with the Gaussian nested mapping method of Xu et al. [33], internal ablation analysis, comparison with existing BPA generation methods, comparison with classical machine learning classifiers, robustness evaluation, and discussion of the experimental results. In the ablation study, four variants, namely Gaussian + Deng entropy, Gaussian + RB divergence, OTSU + RB divergence, and OTSU + Deng entropy, are compared to examine the effects of the OTSU-based grouping structure and the Deng-entropy-based confidence regulation mechanism.

### 5.1. Comparison with the Gaussian Nested Mapping Structure

To examine the feasibility of the proposed OTSU-based grouping mapping structure, a comparative experiment is conducted against the classical Gaussian nested mapping method proposed by Xu et al. [33]. In this experiment, the two methods use the same initial Gaussian support modeling strategy. The main difference lies in the focal element construction mechanism. The Gaussian nested mapping method constructs nested focal elements according to the descending order of support values, whereas the OTSU-based method partitions the ordered support sequence into two non-overlapping focal elements. Other experimental settings, including the evidence fusion rule and decision-making procedure, are kept the same for a fair comparison.

Table 3 reports the average classification accuracy of the two mapping structures. As shown in Table 3, the OTSU grouping mapping structure obtains higher accuracy on four of the five datasets, with a clear improvement on Haberman. However, the Gaussian nested mapping structure performs better on Wine, indicating that the advantage of OTSU grouping is dataset-dependent. These results suggest that OTSU grouping can serve as a feasible alternative to nested focal element construction. By partitioning the ordered support values into non-overlapping groups, it may reduce unnecessary uncertainty introduced by repeated nesting in some cases.

### 5.2. Internal Ablation Study on BPA Generation and Uncertainty Quantification

To examine the effects of the main components in the proposed framework, an internal ablation study is conducted. Four BPA generation variants are compared, namely Gaussian + Deng Ent, Gaussian + Reinforced Belief Divergence (RB divergence [62]), OTSU + RB divergence, and ODE-BPA. In this comparison, ODE-BPA corresponds to the combination of OTSU-based grouping mapping and Deng-entropy-based confidence regulation. The results are reported in Table 4.

As shown in Table 4, ODE-BPA obtains the highest accuracy on 17 of the twenty-five datasets. On the remaining datasets, ODE-BPA still shows comparable performance. For example, on Voting, the difference between ODE-BPA and the best-performing method is small. However, on Wine, OTSU + RB divergence achieves a higher accuracy than ODE-BPA. This indicates that while the proposed combination is generally effective, its performance can vary depending on the data distribution. Table 5 further summarizes the average metrics for each method. ODE-BPA achieves the highest average accuracy (85.49%) and the best average rank (1.70), along with the lowest average standard deviation (4.03%).

To assess the statistical significance of these results, the Friedman test and Iman–Davenport correction are applied. Both tests clearly reject the null hypothesis ($F_{F}=7.289$, p<0.001), indicating significant differences among the compared methods. Subsequently, post hoc Wilcoxon signed-rank tests with Holm correction are performed using ODE-BPA as the control method. In this analysis, Rank Difference measures the gap in average ranks between ODE-BPA and the compared variant; the Raw *p*-value represents the direct significance level from the Wilcoxon test; and the Holm-adjusted *p*-value provides a conservative estimate by controlling the family-wise error rate in multiple comparisons. The results are summarized in Table 6.

As shown in Table 6, all Holm-adjusted *p*-values remain below 0.05, indicating that the observed differences in performance are statistically significant. These results suggest that the integration of OTSU grouping and Deng-entropy-based regulation consistently contributes to the classification performance, although the final effectiveness is still influenced by dataset-specific characteristics.

### 5.3. Comparison with Existing BPA Generation Methods

To further evaluate the effectiveness of the proposed method, ODE-BPA is compared with several existing BPA generation methods. The compared methods include Wang’s Gaussian-BPA [34], Xu’s Gaussian-BPA [33], Jiang’s FuzzyEnv-BPA [39], Li’s RBFN-BPA [63], and Kang’s Interval-BPA [38]. Among them, Wang’s Gaussian-BPA and Xu’s Gaussian-BPA are both based on Gaussian distribution modeling, while they differ in the specific BPA mapping mechanism. Jiang’s FuzzyEnv-BPA constructs BPAs under a fuzzy environment. Li’s RBFN-BPA determines basic belief assignments using a radial basis function network. Kang’s Interval-BPA generates BPAs based on interval numbers, where its support coefficient is set to α=5. The classification results over 100 independent runs are shown in Table 7.

As shown in Table 7, ODE-BPA yields the highest mean accuracy on 13 of the 25 datasets, demonstrating competitive performance across diverse classification scenarios. However, its effectiveness is not universally superior. For instance, Wang’s Gaussian-BPA performs better on Fire and Early Diabetes, Xu’s Gaussian-BPA achieves better results on Acute Inflammations and Wine, while Li’s RBFN-BPA and Kang’s Interval-BPA stand out on Planning and Immunotherapy, respectively. This suggests that the performance of these BPA generation methods remains highly sensitive to the inherent data distribution and feature characteristics of different tasks.

To summarize the overall performance, Table 8 reports the mean accuracy, standard deviation across datasets, average rank, and best-performing count of each method. As presented in Table 8, ODE-BPA ranks first in both average accuracy (85.51%) and average rank (1.84) among the six methods. It is worth noting that the gap in mean accuracy between ODE-BPA and Wang’s Gaussian-BPA is narrow, although ODE-BPA achieves a slightly better average rank and a higher best count.

To rigorously examine whether the differences among the compared methods are statistically meaningful, the Friedman test with Iman–Davenport correction is conducted. The test yields $F_{F}=23.73$ with a *p*-value of $1.11\times 10^{-16}$ (p<0.05), completely rejecting the null hypothesis of equivalent performance among the six BPA generation methods. Given this overall significance, post hoc Wilcoxon signed-rank tests with Holm correction are performed by taking ODE-BPA as the reference method. The statistical comparisons are reported in Table 9.

As reported in Table 9, the Holm-adjusted *p*-values for the comparisons between ODE-BPA and Kang’s Interval-BPA, Li’s RBFN-BPA, Jiang’s FuzzyEnv-BPA, and Xu’s Gaussian-BPA all remain below the 0.05 threshold. This indicates that ODE-BPA provides a statistically significant improvement over these four methods. However, the statistical comparison between ODE-BPA and Wang’s Gaussian-BPA yields an adjusted *p*-value of 0.3643. This reveals that, despite having a better average rank, ODE-BPA does not demonstrate a statistically significant advantage over Wang’s Gaussian-BPA across the evaluated datasets.

In summary, while ODE-BPA serves as a highly robust and competitive alternative for BPA generation, its practical advantage over state-of-the-art Gaussian distribution models is incremental and fundamentally dependent on the specific characteristics of the data.

### 5.4. Comparison with Classical Machine Learning Classifiers

To evaluate the proposed method from a broader classification perspective, ODE-BPA is compared with four classical machine learning classifiers, namely K-nearest neighbors (KNN), support vector machine (SVM), decision tree, and naive Bayes. These classifiers are implemented using standard settings in MATLAB R2025a. Specifically, KNN uses five nearest neighbors with feature standardization, SVM employs a Gaussian kernel under the ECOC framework, the decision tree uses the default CART algorithm, and naive Bayes applies the default distribution setting. When the default naive Bayes model encounters numerical issues, kernel density estimation is used as a fallback.

The comparison is conducted on twenty-five benchmark datasets. The same repeated cross-validation protocol is adopted, and the mean classification accuracy and standard deviation are reported in Table 10.

As shown in Table 10, ODE-BPA obtains the best accuracy on Iris, Haberman, and Lenses, and ties for the best result on Mesothelioma and Blood Transfusion. On several datasets, such as Voting, Divorce, and Fire, its accuracy is close to the leading classical classifier. However, classical machine learning methods perform better on some other datasets. For example, SVM performs strongly on Planning, Monk1, Vertebral Column, and Banknote, while the decision tree achieves high accuracy on Voting, Fire, Knowledge, and Early Diabetes. These results indicate that ODE-BPA does not universally outperform classical classifiers, but it remains competitive on several datasets. To summarize the overall performance, Table 11 reports the mean accuracy, the standard deviation of accuracy across datasets, the average rank, and the number of datasets on which each method achieves thebest result.

As shown in Table 11, the decision tree achieves the highest mean accuracy and the largest best count, while KNN obtains the best average rank. ODE-BPA ranks fourth among the five compared methods. Its average rank is close to that of SVM and better than that of naive Bayes. In terms of best count, ODE-BPA achieves or ties for the best result on five datasets. This suggests that ODE-BPA provides comparable, but not dominant, performance when compared with classical machine learning classifiers under standard settings. To examine whether the observed rank differences are statistically meaningful, the Friedman test and the Iman–Davenport correction are conducted. The Friedman test yields $\chi_{F}^{2}=5.624$ and a *p*-value of 0.229. The Iman–Davenport correction further gives $F_{F}=1.430$ and a *p*-value of 0.230. Since both *p*-values are larger than 0.05, the null hypothesis of equivalent average ranks cannot be rejected at the 0.05 significance level. This indicates that the performance differences among the five classifiers are not statistically significant over the tested datasets. For completeness, a rank-based Holm post hoc procedure is also performed using ODE-BPA as the reference method. The rank difference is computed as the average rank of the compared method minus the average rank of ODE-BPA. The results are reported in Table 12.

As shown in Table 12, none of the Holm-adjusted *p*-values is below 0.05. Therefore, no statistically significant pairwise rank difference is observed between ODE-BPA and the selected classical classifiers. In terms of average rank, ODE-BPA is numerically lower than KNN, but the corresponding Holm-adjusted *p*-value is 0.663, indicating that this difference is not statistically significant. Meanwhile, ODE-BPA shows similar rank performance to SVM, naive Bayes, and decision, with Holm-adjusted *p*-values of 1.000 in all comparisons. These results indicate that ODE-BPA can achieve classification performance comparable to some classical classifiers while retaining the interpretability and uncertainty modeling capability of the D–S theory framework. Nevertheless, its relative performance remains dependent on the data distribution and feature characteristics of each dataset.

### 5.5. Robustness Evaluation Under Noise and Conflicting Evidence

To further examine the behavior of ODE-BPA under disturbed conditions, a robustness evaluation is conducted on the Haberman and Iris datasets. In this experiment, both feature noise and conflicting evidence are introduced into the test samples. The Gaussian-based BPA generation method is used as the baseline, and ODE-BPA is evaluated under different noise levels.

For each test sample, Gaussian noise is added to the feature values according to

(18) $$x_{j}^{\prime }=x_{j}+\sigma \cdot s_{j}\cdot \epsilon ,$$

where $x_{j}$ is the original value of the *j*-th feature, $s_{j}$ is the standard deviation of this feature in the training set, and ϵ follows the standard normal distribution. The noise levels are set to σ=0, 0.05, 0.10, 0.15, and 0.20.

Conflicting evidence is then injected into one selected feature at a time. For each target feature, a certain proportion of test samples is randomly selected according to the conflict ratio. The conflict ratio varies from 0% to 100%, with an interval of 10%. For these selected samples, the original noisy support vector $A_{n}$ is mixed with an artificially generated conflicting support vector $A_{c}$:

(19) $$A_{f}=\left(1-\lambda \right)A_{n}+\lambda A_{c}$$

where λ=0.50 in this experiment. Thus, the noisy evidence and conflicting evidence are assigned equal weights. The conflicting support vector is designed to assign larger support to incorrect classes, thereby creating misleading local evidence.

The disturbance is applied separately to each feature, and each subfigure reports the result when one specific feature is disturbed. For clarity, the Gaussian baseline is shown under the moderate noise level σ=0.10, while ODE-BPA is shown under all tested noise levels.

**Figure 3:**
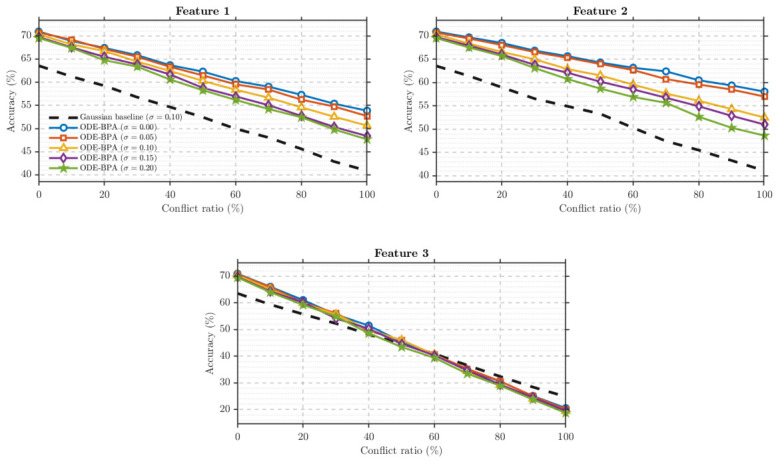
Robustness evaluation on the Haberman dataset under different conflict ratios and noise levels.

The results on the Haberman dataset are shown in Figure 3. Since Haberman contains three features, three subfigures are presented. Each subfigure corresponds to the case where one specific feature is disturbed by conflicting evidence.

As shown in Figure 3, the accuracy generally decreases as the conflict ratio increases, indicating that the injected conflicting evidence affects the fusion result. For Feature 1 and Feature 2, ODE-BPA usually performs better than the Gaussian baseline, especially under higher conflict ratios. However, when Feature 3 is disturbed, the accuracy of ODE-BPA drops rapidly. This suggests that the robustness of ODE-BPA is related to the importance of the disturbed feature.

**Figure 4:**
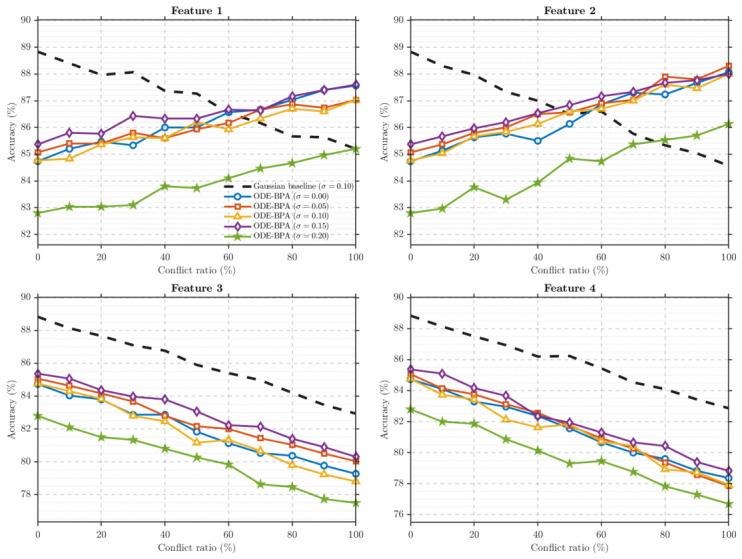
Robustness evaluation on the Iris dataset under different conflict ratios and noise levels.

The results on the Iris dataset are shown in Figure 4. Since Iris contains four features, four subfigures are presented. The same disturbance mechanism is applied to each feature separately.

As shown in Figure 4, ODE-BPA shows better resistance to conflict when Feature 1 or Feature 2 is disturbed. In contrast, when Feature 3 or Feature 4 is disturbed, its accuracy decreases as the conflict ratio increases. This may be because these two features contain more discriminative information for the Iris dataset. Therefore, the robustness of ODE-BPA is not only affected by the conflict ratio, but also by the discriminative role of the disturbed feature.

To provide a broader view of the robustness across different data distributions, Table 13 summarizes the accuracy of both the Gaussian baseline and ODE-BPA on seven datasets. This comparison is conducted under a moderate noise level (σ=0.10) when the first feature is disturbed by varying proportions of conflicted test samples.

Table 13 provides a cross-dataset comparison under a fixed moderate noise level, i.e., σ=0.10. Unlike Figure 3 and Figure 4, which show the joint influence of different noise levels and conflict ratios, this table focuses on the effect of increasing conflict when moderate Gaussian noise is present. ODE-BPA consistently outperforms the Gaussian baseline on Haberman and Lenses, and shows better performance under higher conflict ratios on Cryotherapy and Iris. However, the Gaussian baseline remains better on Vertebral Column and Seeds, and performs better than ODE-BPA under stronger conflict on Immunotherapy. These results indicate that ODE-BPA can improve robustness to conflicting evidence under noisy conditions in several cases, but its advantage remains feature-dependent and dataset-specific.

### 5.6. Discussion

This subsection briefly discusses two issues related to the proposed ODE-BPA framework: the use of binary OTSU and the role of Deng entropy.

First, ODE-BPA adopts a binary OTSU strategy to divide the ordered support sequence into two non-overlapping focal element groups. This design is simple, parameter-free, and relatively stable, especially when the number of classes is small. However, binary partitioning may be insufficient when the support values contain more than two clearly separated regions. In such cases, multi-threshold OTSU could be considered to generate more refined focal element structures. Nevertheless, this may also increase the number of focal elements and the computational cost of evidence fusion. Therefore, binary OTSU is used in this paper as a conservative choice, while multi-threshold OTSU is left for future work.

Second, Deng entropy is used in ODE-BPA to derive the confidence coefficient for mass allocation, because it reflects both the dispersion of mass values and the cardinalities of focal elements. This feature is compatible with the grouped focal elements generated by OTSU. Nevertheless, Deng entropy is not treated in this work as a flawless or universally axiomatized total uncertainty measure. Previous study has discussed several mathematical requirements for uncertainty measures in D–S theory [64], and Abellán [65] provided a systematic analysis of Deng entropy from this perspective. Specifically, Deng entropy satisfies probabilistic consistency when all focal elements are singletons, because it reduces to Shannon entropy in this special case. However, it does not strictly satisfy several standard requirements for total uncertainty measures, including set consistency, range, subadditivity, additivity, and monotonicity.

In the proposed ODE-BPA framework, the influence of these limitations should be understood according to the specific role of Deng entropy. As defined in Equation (16), the Deng entropy is normalized by the maximum Deng entropy under the same focal-element cardinality configuration. Therefore, the confidence coefficient is defined on a relative scale and is bounded in [0,1]. This design does not remove the theoretical limitations related to set consistency or range, but it reduces the direct dependence of mass allocation on the absolute value of Deng entropy. Similarly, ODE-BPA employs Deng entropy to evaluate the uncertainty of an individual feature-level BPA before evidence combination, rather than to measure uncertainty over a joint or multidimensional product space. Hence, the violations of additivity and subadditivity have limited direct influence on the generation of individual BPAs in the current framework. By contrast, the lack of monotonicity is more directly related to ODE-BPA. As discussed in [65], Deng entropy may assign a larger uncertainty value to a BPA that is intuitively more informative under certain BPA configurations. Since a larger Deng entropy leads to a smaller confidence coefficient in Equation (16), the entropy-derived confidence regulation may inherit this potential counterintuitive behavior.

The structural interpretation of Deng entropy also requires caution. Abellán [65] pointed out two structural issues in its formulation. First, the Shannon-like discord term and the cardinality-based non-specificity term have different ranges. As the size of the frame of discernment increases, the non-specificity component may dominate the total entropy value, making the discord component relatively less influential. Second, the Shannon-like discord term can be positive even when all focal elements share a common element, which is usually regarded as a situation with no genuine discord in D–S theory. In ODE-BPA, the latter issue has limited direct relevance because Deng entropy is computed on the OTSU-induced two-focal-element structure, where the two constructed focal elements $F_{1}$ and $F_{2}$ are mutually disjoint. Nevertheless, the imbalance between the discord component and the non-specificity component remains relevant to the uncertainty quantification process, because it may affect the resulting confidence coefficient.

Based on the above analysis, the non-monotonic behavior of Deng entropy and the structural imbalance between its discord and non-specificity components are the most relevant theoretical issues for ODE-BPA. These issues may influence the confidence assigned to the induced focal-element structure in some specific cases. Therefore, Deng entropy is regarded in this paper as a practical uncertainty-sensitive indicator for BPA generation, rather than as a strictly rigorous measure of total uncertainty in D–S theory. Future work will explore alternative uncertainty measures with stronger monotonicity and structural consistency, such as upper bounds of uncertainty [66], plausibility entropy [67], and maximum-entropy-based uncertainty measures [68], to improve the theoretical consistency and robustness of the proposed BPA generation framework.

Overall, ODE-BPA provides a competitive BPA generation strategy by combining OTSU-based focal element construction with entropy-guided mass allocation. Future work will investigate multi-threshold OTSU and alternative uncertainty measures to further improve the flexibility and theoretical interpretability of the proposed framework.

## 6. Conclusions

This paper proposes ODE-BPA, a BPA generation method based on OTSU partitioning and Deng entropy within the framework of D-S theory. The method aims to alleviate the structural rigidity that may occur in strictly nested focal element mapping schemes. In ODE-BPA, OTSU partitioning is used to construct non-overlapping focal elements from the ordered support distribution of hypotheses, while Deng entropy is used to regulate the confidence degree in the mass allocation process. This design provides an adaptive way to generate BPAs for evidential classification.

Experiments on twenty-five benchmark datasets show that ODE-BPA is effective within the D–S-based BPA generation framework. The comparison with Gaussian nested mapping and the internal ablation study confirm that both OTSU-based grouping and Deng-entropy-based confidence regulation contribute to focal element construction and mass allocation. Compared with existing BPA generation methods, ODE-BPA achieves the best average accuracy and average rank, with statistically significant improvements over most BPA-based competitors. In addition, comparisons with classical machine learning classifiers show that ODE-BPA provides comparable performance to SVM, naive Bayes, and decision tree under standard settings, although it is numerically weaker than KNN in terms of average rank. This indicates that the main advantage of ODE-BPA lies in uncertainty modeling and evidence fusion within the D–S framework, rather than in replacing general-purpose classifiers. Its relatively limited improvement over classical classifiers may be related to the binary OTSU grouping strategy and the theoretical limitations of Deng-entropy-based confidence regulation. The robustness experiments further show that ODE-BPA can alleviate local noisy and conflicting evidence in some cases, but its effectiveness remains dependent on dataset characteristics, disturbed features, and evidence structure.

## Figures and Tables

**Table 1:** Comparison of the BPAs generated by ODE-BPA and the traditional nested mapping method.

Item	ODE-BPA	Nested Mapping Method
Dominant singleton focal element	$\left\{\theta_{3}\right\}$	$\left\{\theta_{3}\right\}$
Mass of dominant singleton	0.3262	0.7200
Main non-singleton focal elements	$\left\{\theta_{1},\theta_{2},\theta_{4}\right\}$	$\left\{\theta_{3},\theta_{1}\right\}$, $\left\{\theta_{3},\theta_{1},\theta_{2}\right\}$
Mass assigned to Θ	0.5470	0.0500
Number of focal elements	3	4
Nested focal structure	No	Yes
Deng entropy of focal structure	1.6410	1.9171

**Table 2:** Basic information of the benchmark datasets used in the experiments.

Dataset	Number of Samples	Number of Features	Number of Classes
Cryotherapy	90	6	2
Immunotherapy	90	7	2
Acute Inflammations	120	6	2
Divorce	170	54	2
Planning	182	12	2
Fire	244	13	2
Haberman	306	3	2
Vertebral Column (2C)	310	6	2
Mesothelioma	324	34	2
Voting	435	16	2
Wholesale Customers	440	7	2
Early Diabetes	520	16	2
Monk1	556	6	2
WDBC	568	30	2
Australian	690	14	2
Blood Transfusion	748	5	2
Fourclass	861	2	2
Raisin	900	7	2
Banknote	1372	4	2
Lenses	24	4	3
Iris	150	4	3
Wine	178	13	3
Seeds	210	7	3
Soybean	47	35	4
Knowledge	403	5	4

**Table 3:** Average classification accuracy (%) of different focal element mapping structures.

Mapping Structure	Iris	Seeds	Wine	Australian	Haberman
Gaussian nested mapping	94.69	90.07	**97.27**	80.33	61.30
OTSU grouping mapping	**95.16**	**90.15**	96.51	**80.35**	**75.03**

**Note:** Bold values indicate the best classification accuracy.

**Table 4:** Classification accuracy (%) of the internal ablation study over 100 independent runs.

Dataset	Gaussian + Deng Ent	Gaussian + RB Diverg	OTSU + RB Diverg	ODE-BPA
Cryotherapy	84.00 ± 8.15	81.35 ± 8.64	83.94 ± 8.05	**85.38 ± 7.70**
Immunotherapy	55.99 ± 12.55	51.82 ± 12.47	56.69 ± 12.87	**59.16 ± 13.67**
Acute Inflammations	97.17 ± 3.61	**98.25 ± 3.38**	96.00 ± 4.37	97.25 ± 3.53
Divorce	97.38 ± 2.78	97.05 ± 2.82	96.98 ± 2.89	**97.45 ± 2.71**
Planning	44.13 ± 7.18	42.82 ± 7.50	44.27 ± 6.87	**45.29 ± 7.21**
Fire	**94.46 ± 2.67**	93.63 ± 2.78	94.45 ± 2.67	94.16 ± 2.73
Haberman	73.75 ± 4.57	61.19 ± 6.51	75.03 ± 4.23	**75.36 ± 3.97**
Vertebral Column (2C)	78.32 ± 4.57	76.58 ± 4.48	78.01 ± 4.63	**79.78 ± 4.51**
Mesothelioma	**100.00 ± 0.00**	**100.00 ± 0.00**	**100.00 ± 0.00**	**100.00 ± 0.00**
Voting	94.30 ± 2.12	**94.41 ± 2.19**	94.06 ± 2.21	94.36 ± 2.18
Wholesale Customers	89.62 ± 3.01	89.56 ± 2.91	89.61 ± 3.02	**89.72 ± 2.73**
Early Diabetes	88.70 ± 2.71	84.00 ± 3.31	**89.23 ± 2.98**	88.34 ± 2.60
Monk1	**66.87 ± 3.99**	58.94 ± 4.05	66.51 ± 3.96	66.67 ± 4.04
WDBC	92.60 ± 2.46	93.12 ± 2.56	**93.14 ± 2.49**	92.35 ± 2.47
Australian	80.08 ± 2.94	80.22 ± 2.93	80.23 ± 2.89	**80.54 ± 2.91**
Blood Transfusion	**100.00 ± 0.00**	**100.00 ± 0.00**	**100.00 ± 0.00**	**100.00 ± 0.00**
Fourclass	**71.82 ± 3.09**	**71.82 ± 3.09**	**71.82 ± 3.09**	**71.82 ± 3.09**
Raisin	83.69 ± 2.52	83.28 ± 2.37	83.88 ± 2.44	**84.58 ± 2.49**
Banknote	84.48 ± 2.34	83.89 ± 2.26	84.42 ± 2.22	**84.56 ± 2.27**
Lenses	87.37 ± 11.30	87.42 ± 11.38	82.83 ± 13.70	**87.46 ± 11.33**
Iris	95.43 ± 3.56	95.39 ± 3.54	95.28 ± 3.49	**95.87 ± 3.23**
Wine	93.16 ± 3.59	93.73 ± 3.44	**96.14 ± 2.97**	93.27 ± 3.73
Seeds	89.67 ± 4.19	88.82 ± 4.43	90.35 ± 3.99	**90.93 ± 3.92**
Soybean	59.42 ± 5.07	55.24 ± 4.38	76.58 ± 4.15	**97.80 ± 4.30**
Knowledge	83.97 ± 3.56	83.36 ± 3.65	**85.19 ± 3.60**	85.05 ± 3.53

**Note:** Bold values indicate the best classification accuracy among the compared methods.

**Table 5:** Average performance and ranking of the four ablation variants over 25 datasets.

Method	Average Accuracy	Average Std.	Average Rank	Best Count
ODE-BPA	**85.486**	**4.034**	**1.700**	**17**
OTSU + RB Diverg	83.786	4.151	2.500	7
Gaussian + Deng Ent	83.455	4.101	2.580	5
Gaussian + RB Diverg	81.836	4.203	3.220	5

**Note:** Bold values indicate the best performance metrics among the compared variants.

**Table 6:** Post hoc Wilcoxon signed-rank test with Holm correction using ODE-BPA as the reference method.

Comparison	Rank Difference	Raw *p*-Value	Holm-Adjusted *p*-Value
ODE-BPA vs. Gaussian + RB Diverg	1.520	$3.15\times 10^{-5}$	$9.44\times 10^{-5}$
ODE-BPA vs. Gaussian + Deng Ent	0.880	0.0160	0.0319
ODE-BPA vs. OTSU + RB Diverg	0.800	0.0285	0.0319

**Table 7:** Classification accuracy (%) of ODE-BPA and existing BPA generation methods over 100 independent runs.

Dataset	Wang’s Gaussian	Xu’s Gaussian	Jiang’s FuzzyEnv	Li’s RBFN	Kang’s Interval	ODE-BPA
Cryotherapy	84.01 ± 8.14	82.16 ± 8.23	80.30 ± 8.45	56.35 ± 5.19	55.34 ± 5.52	**85.42 ± 8.39**
Immunotherapy	56.33 ± 13.88	54.44 ± 12.10	46.78 ± 12.39	28.11 ± 9.30	**78.11 ± 3.60**	59.11 ± 13.11
Acute Inflammations	96.75 ± 3.77	**98.25 ± 2.91**	96.88 ± 3.47	58.33 ± 0.00	96.67 ± 3.35	97.58 ± 3.27
Divorce	96.91 ± 2.97	96.91 ± 2.97	96.97 ± 2.63	86.59 ± 20.46	3.00 ± 2.13	**97.32 ± 2.93**
Planning	44.45 ± 7.10	42.60 ± 8.18	50.58 ± 8.10	**59.51 ± 8.01**	36.37 ± 16.52	45.94 ± 7.13
Fire	**94.14 ± 2.84**	93.79 ± 2.79	93.44 ± 3.31	43.44 ± 0.81	80.46 ± 5.58	94.00 ± 2.81
Haberman	74.79 ± 4.28	60.87 ± 6.07	70.44 ± 7.08	74.54 ± 2.94	60.15 ± 15.96	**74.97 ± 4.27**
Vertebral Column (2C)	77.84 ± 4.21	77.03 ± 4.22	68.53 ± 6.03	41.53 ± 16.92	32.74 ± 1.33	**79.87 ± 3.80**
Mesothelioma	**100.00 ± 0.00**	**100.00 ± 0.00**	32.82 ± 3.55	70.37 ± 0.60	36.59 ± 12.67	**100.00 ± 0.00**
Voting	94.38 ± 1.85	94.34 ± 1.81	88.89 ± 2.90	94.06 ± 2.32	38.48 ± 0.53	**94.43 ± 1.95**
Wholesale Customers	**90.11 ± 3.06**	89.82 ± 2.98	87.65 ± 3.32	81.38 ± 4.42	44.14 ± 16.36	89.86 ± 3.36
Early Diabetes	**88.58 ± 2.73**	84.50 ± 3.41	74.96 ± 3.05	52.65 ± 6.21	60.75 ± 1.55	88.41 ± 2.96
Monk1	66.56 ± 3.96	62.96 ± 4.11	66.67 ± 4.01	**66.86 ± 4.13**	66.67 ± 4.01	66.67 ± 4.01
WDBC	93.28 ± 2.16	**93.36 ± 2.31**	91.69 ± 2.52	86.72 ± 2.94	69.75 ± 2.79	92.45 ± 2.32
Australian	80.38 ± 2.82	80.31 ± 2.87	77.14 ± 3.44	55.94 ± 0.68	44.88 ± 2.21	**80.91 ± 3.29**
Blood Transfusion	**100.00 ± 0.00**	**100.00 ± 0.00**	56.20 ± 7.17	76.20 ± 0.25	1.00 ± 10.00	**100.00 ± 0.00**
Fourclass	71.78 ± 3.04	71.78 ± 3.04	**73.82 ± 3.57**	73.62 ± 3.14	42.25 ± 10.97	71.78 ± 3.04
Raisin	83.69 ± 2.09	83.41 ± 2.14	77.37 ± 3.48	76.86 ± 2.45	60.17 ± 2.74	**84.55 ± 2.05**
Banknote	84.49 ± 2.17	84.06 ± 2.23	80.29 ± 2.37	78.52 ± 2.01	67.27 ± 2.11	**84.60 ± 2.07**
Lenses	85.00 ± 11.44	85.00 ± 11.44	62.50 ± 7.26	16.67 ± 0.00	41.67 ± 21.38	**87.50 ± 12.01**
Iris	95.37 ± 3.64	94.57 ± 3.69	94.37 ± 3.81	90.23 ± 4.62	93.60 ± 4.23	**95.83 ± 3.36**
Wine	97.11 ± 2.66	**97.33 ± 2.31**	96.38 ± 3.22	65.40 ± 5.38	85.88 ± 4.62	93.07 ± 3.73
Seeds	90.38 ± 4.37	90.05 ± 4.39	89.74 ± 4.42	90.24 ± 4.44	86.14 ± 5.71	**90.67 ± 4.14**
Soybean	95.73 ± 5.72	55.21 ± 4.75	21.33 ± 1.09	21.33 ± 1.09	59.46 ± 4.85	**97.88 ± 4.27**
Knowledge	**88.50 ± 3.55**	83.46 ± 3.61	85.91 ± 4.79	76.67 ± 5.16	38.95 ± 5.33	84.92 ± 3.92

**Note:** Bold values indicate the best classification accuracy among the compared methods.

**Table 8:** Average performance and ranking of existing BPA generation methods over 25 datasets.

Method	Mean Accuracy	Std. Accuracy	Average Rank	Best Count
ODE-BPA	**85.510**	13.310	**1.840**	**13**
Wang’s Gaussian-BPA	85.222	13.813	2.320	5
Xu’s Gaussian-BPA	82.248	15.899	3.160	4
Jiang’s FuzzyEnv-BPA	74.466	20.265	3.820	1
Li’s RBFN-BPA	64.885	21.516	4.580	2
Kang’s Interval-BPA	55.220	24.861	5.280	1

**Note:** Bold values indicate the best performance metrics among the compared methods.

**Table 9:** Post hoc Wilcoxon signed-rank test with Holm correction using ODE-BPA as the reference method.

Comparison	Rank Difference	Raw *p*-Value	Holm-Adjusted *p*-Value
ODE-BPA vs. Kang’s Interval-BPA	3.440	$7.98\times 10^{-11}$	$3.99\times 10^{-10}$
ODE-BPA vs. Li’s RBFN-BPA	2.740	$2.24\times 10^{-7}$	$8.97\times 10^{-7}$
ODE-BPA vs. Jiang’s FuzzyEnv-BPA	1.980	$1.83\times 10^{-4}$	$5.48\times 10^{-4}$
ODE-BPA vs. Xu’s Gaussian-BPA	1.320	0.0126	0.0252
ODE-BPA vs. Wang’s Gaussian-BPA	0.480	0.3643	0.3643

**Table 10:** Classification accuracy (%) of ODE-BPA and classical machine learning classifiers.

Dataset	KNN	SVM	Decision Tree	Naive Bayes	ODE-BPA
Cryotherapy	88.39 ± 6.88	85.84 ± 7.68	88.39 ± 7.09	83.97 ± 8.27	84.92 ± 7.45
Immunotherapy	80.94 ± 5.19	78.06 ± 3.20	81.83 ± 8.43	78.89 ± 9.24	59.61 ± 13.14
Acute Inflammations	**100.00 ± 0.00**	**100.00 ± 0.00**	99.92 ± 0.83	95.29 ± 5.54	97.56 ± 3.23
Divorce	97.65 ± 2.33	71.15 ± 5.83	96.50 ± 2.98	**97.74 ± 2.36**	97.41 ± 2.59
Planning	62.93 ± 6.37	**73.43 ± 2.17**	57.58 ± 8.03	64.07 ± 6.09	45.30 ± 8.02
Fire	90.55 ± 3.86	83.50 ± 4.68	**97.40 ± 2.52**	93.60 ± 3.24	94.13 ± 3.44
Haberman	70.05 ± 3.74	73.11 ± 3.21	68.41 ± 4.38	74.67 ± 3.23	**75.25 ± 3.99**
Vertebral Column (2C)	81.06 ± 4.71	**83.29 ± 4.41**	79.56 ± 5.01	77.76 ± 4.63	79.77 ± 4.48
Mesothelioma	86.17 ± 3.72	70.37 ± 0.60	**100.00 ± 0.00**	69.21 ± 3.07	**100.00 ± 0.00**
Voting	93.28 ± 2.62	80.82 ± 3.01	**94.74 ± 2.31**	94.21 ± 2.51	94.38 ± 2.53
Wholesale Customers	**90.05 ± 2.96**	88.56 ± 2.91	89.14 ± 3.43	89.72 ± 2.76	89.72 ± 2.79
Early Diabetes	92.37 ± 2.50	93.31 ± 2.15	**93.41 ± 2.36**	88.97 ± 3.06	88.47 ± 2.75
Monk1	88.23 ± 5.92	**95.14 ± 2.35**	88.67 ± 8.26	66.50 ± 3.83	66.67 ± 3.73
WDBC	**96.58 ± 1.67**	63.15 ± 0.54	92.94 ± 2.22	93.36 ± 1.95	92.43 ± 2.11
Australian	**84.30 ± 2.41**	73.21 ± 3.37	82.88 ± 3.09	80.20 ± 2.58	80.76 ± 3.00
Blood Transfusion	99.73 ± 0.39	99.81 ± 0.41	**100.00 ± 0.00**	80.81 ± 3.39	**100.00 ± 0.00**
Fourclass	**99.99 ± 0.08**	99.38 ± 0.56	97.88 ± 1.11	75.51 ± 2.73	71.72 ± 3.02
Raisin	85.03 ± 2.27	**86.41 ± 2.21**	81.83 ± 2.87	83.79 ± 2.28	84.56 ± 2.12
Banknote	99.84 ± 0.21	**99.93 ± 0.15**	98.06 ± 0.87	83.90 ± 2.06	84.52 ± 1.93
Lenses	77.80 ± 19.21	63.00 ± 6.03	79.35 ± 22.33	63.00 ± 6.03	**87.55 ± 13.97**
Iris	94.87 ± 3.68	95.03 ± 3.83	94.93 ± 3.37	95.23 ± 3.80	**95.93 ± 3.13**
Wine	96.20 ± 2.98	51.27 ± 5.07	90.67 ± 4.90	**97.11 ± 2.75**	92.97 ± 3.55
Seeds	92.50 ± 3.52	**93.31 ± 3.40**	91.07 ± 3.95	90.36 ± 4.37	90.76 ± 4.48
Soybean	**100.00 ± 0.00**	36.00 ± 3.28	96.79 ± 6.03	99.79 ± 1.49	97.66 ± 4.44
Knowledge	83.45 ± 3.69	90.79 ± 3.02	**92.06 ± 2.76**	89.27 ± 2.98	84.77 ± 3.47

**Note:** Bold values indicate the best classification accuracy among the compared methods.

**Table 11:** Average performance and ranking of ODE-BPA and classical classifiers.

Method	Mean Acc.	Std. Acc.	Avg. Rank	Best Count
KNN	89.278	**9.634**	**2.520**	7
Decision Tree	**89.360**	10.328	2.740	**8**
SVM	81.115	16.174	3.120	7
Naive Bayes	84.277	10.961	3.480	2
ODE-BPA	85.473	13.345	3.140	5

**Note:** Bold values indicate the best performance metrics among the compared methods.

**Table 12:** Rank-based Holm post hoc comparison using ODE-BPA as the reference method.

Comparison	Rank Diff.	*z*-Value	Raw *p*-Value	Holm-Adjusted *p*-Value
ODE-BPA vs. KNN	−0.620	1.386	0.166	0.663
ODE-BPA vs. Decision Tree	−0.400	0.894	0.371	1.000
ODE-BPA vs. Naive Bayes	0.340	0.760	0.447	1.000
ODE-BPA vs. SVM	−0.020	0.045	0.964	1.000

**Table 13:** Robustness comparison (Accuracy %) between Gaussian Baseline and ODE-BPA on seven datasets under moderate noise (σ=0.10) when Feature 1 is disturbed.

Dataset	Method	Proportion of Conflicted Test Samples					
		**0%**	**20%**	**40%**	**60%**	**80%**	**100%**
Haberman	Gaussian Baseline	63.58	59.25	54.66	49.95	45.60	40.88
	ODE-BPA	**70.34**	**66.75**	**62.45**	**58.37**	**54.58**	**50.64**
Iris	Gaussian Baseline	**88.83**	**87.97**	**87.37**	**86.53**	85.67	85.20
	ODE-BPA	84.77	85.37	85.60	85.93	**86.70**	**87.03**
Cryotherapy	Gaussian Baseline	**71.33**	70.04	69.21	67.44	66.36	64.74
	ODE-BPA	70.42	**70.20**	**69.92**	**69.13**	**68.85**	**68.51**
Immunotherapy	Gaussian Baseline	58.00	**55.22**	**51.56**	**48.39**	**44.22**	**41.44**
	ODE-BPA	**58.33**	55.00	50.44	47.56	42.61	39.11
Vertebral Column (2C)	Gaussian Baseline	**69.03**	**68.00**	**66.48**	**65.15**	**64.08**	**62.98**
	ODE-BPA	66.60	65.71	64.48	63.42	62.53	61.55
Seeds	Gaussian Baseline	**62.43**	**62.21**	**62.10**	**61.79**	**61.69**	**61.45**
	ODE-BPA	59.21	58.74	58.21	57.69	57.33	56.81
Lenses	Gaussian Baseline	33.96	33.33	33.54	33.54	33.12	32.92
	ODE-BPA	**36.04**	**36.04**	**35.83**	**35.62**	**35.83**	**35.62**

**Note:** Bold values indicate the best accuracy between the compared methods under the specific noise condition.

## Data Availability

Publicly available datasets were analyzed in this study. The data can be found in the UCI Machine Learning Repository at https://archive.ics.uci.edu/ml/index.php (accessed on 22 May 2026).
